# A Novel Technique for Restricted Kinematic Alignment Total Knee Arthroplasty Without Computer-Assisted Devices

**DOI:** 10.7759/cureus.67233

**Published:** 2024-08-19

**Authors:** Yuzuru Sakakibara, Yusuke Yasutani, Akimitsu Oyama, Atsushi Teramoto

**Affiliations:** 1 Orthopedic Surgery, Muroran City General Hospital, Muroran, JPN; 2 Orthopedic Surgery, Sapporo Medical University School of Medicine, Sapporo, JPN

**Keywords:** computer-assisted surgery, osteoarthritis of the knee, restricted kinematic alignment, kinematic alignment, total knee arthroplasty

## Abstract

Kinematic-alignment total knee arthroplasty (KA-TKA) aims to restore natural limb alignment and joint line obliquity, thereby improving patient satisfaction. Restricted KA-TKA (rKA-TKA) addresses abnormal knee anatomies and seeks to replicate natural anatomical structures within safe alignment boundaries. This study introduces a novel device and technique that enables rKA-TKA without computer-assisted surgery (CAS). The new device allows for precise cartilage thickness measurement and adjustment of osteotomy angles, facilitating accurate alignment. A heel-lift technique for tibial osteotomy is presented, offering a reproducible method for determining the osteotomy volume and angle. These innovations make KA and rKA-TKA feasible in any surgical setting, avoiding the high costs and limited availability associated with CAS.

## Introduction

Mechanically aligned total knee arthroplasty (MA-TKA) is the gold standard surgical treatment for knee osteoarthritis, with good long-term outcomes reported. However, some patients who undergo MA-TKA feel their knee is unnatural, and an additional 20% are dissatisfied with the surgical outcome [1,2]. Kinematic-alignment total knee arthroplasty (KA-TKA) has recently been reported to improve patient satisfaction [3-5]. KA-TKA aims to restore constitutional lower limb alignment and joint line obliquity before arthritis. KA-TKA is a joint resurfacing procedure with the advantage of minimal soft tissue release [6-8]. However, concerns remain regarding the reproduction of outliers, pathological joint surface geometry, and ligament balance. Restricted KA-TKA (rKA-TKA) was developed as an alternative to unrestricted KA-TKA in patients with abnormal or pathological knee anatomy after osteoarthritis. The rKA-TKA concept aims to recreate anatomical structures within safe boundaries while avoiding extreme varus-valgus alignment [9,10]. Meticulous preoperative planning is required to predict a patient's anatomy and ligament balance. Furthermore, actual ligament balance can only be confirmed intraoperatively. Hence, rKA-TKA requires anatomical modifications to fit within these boundaries and is ideally performed using computer-assisted surgery (CAS), such as patient-specific instrumentation (PSI), computer navigation, or robotic assistance [11]. However, CAS, such as navigation and robotics, is only available in certain institutions and is costly to implement and maintain. In this study, we propose a device and novel technique that can be used for unrestricted KA and rKA-TKA without CAS. The devices and methods presented here make it possible to perform rKA-TKA at any institution.

## Technical report

Preoperative planning

The lateral distal femoral angle (LDFA) and medial proximal tibial angle (MPTA) were measured using preoperative long-leg radiographs. The arithmetic hip-knee-ankle angle (aHKA) was calculated by subtracting the LDFA from the MPTA. The femur can be osteotomized to the thickness of the component using the calibrated KA-TKA technique with an intramedullary rod, thus preserving femoral anatomy. Preoperative boundary planning is necessary in cases of abnormal LDFA values due to hypoplasia of the lateral condyle or excessive varus and valgus deformities resulting from bone loss. We set the aHKA from -5° to +3°, the LDFA from 85° to 95°, and the MPTA from 85° to 90° based on previous reports [2,10,12].

Surgical techniques

Distal Femoral Osteotomy

**Figure 1:**
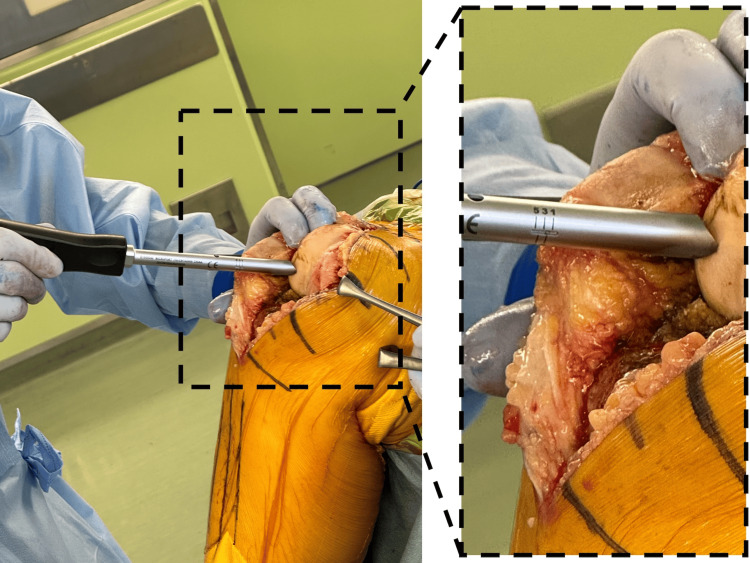
Articular cartilage measuring instrument It is possible to measure cartilage thickness by pressing against the distal femoral cartilage. The image shows a scale of 2 mm.

We use a cartilage thickness gauge (MicroPort®, Memphis, USA), a unique device to measure cartilage thickness in the distal femur (Figure 1).

**Figure 2:**
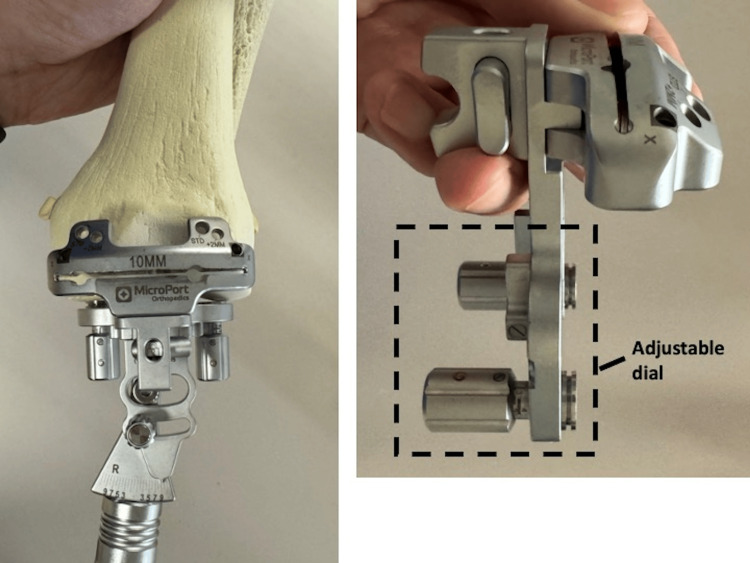
The paddle that contacts the distal femur is equipped with a dial that adjusts for the measured cartilage

The cartilage thickness is measured by pressing the device against its surface. The paddle contacting the distal femur has a dial adjusted for the measured cartilage thickness (Figure 2).

The dial is adjusted medially and laterally to account for the thickness of the implant. If there is significant bone loss or severe malalignment, the osteotomy angle can be adjusted using an angle scale as a reference. In such cases, the angle can be easily modified using a dial on the paddle.

Heel Lift Technique

**Figure 3:**
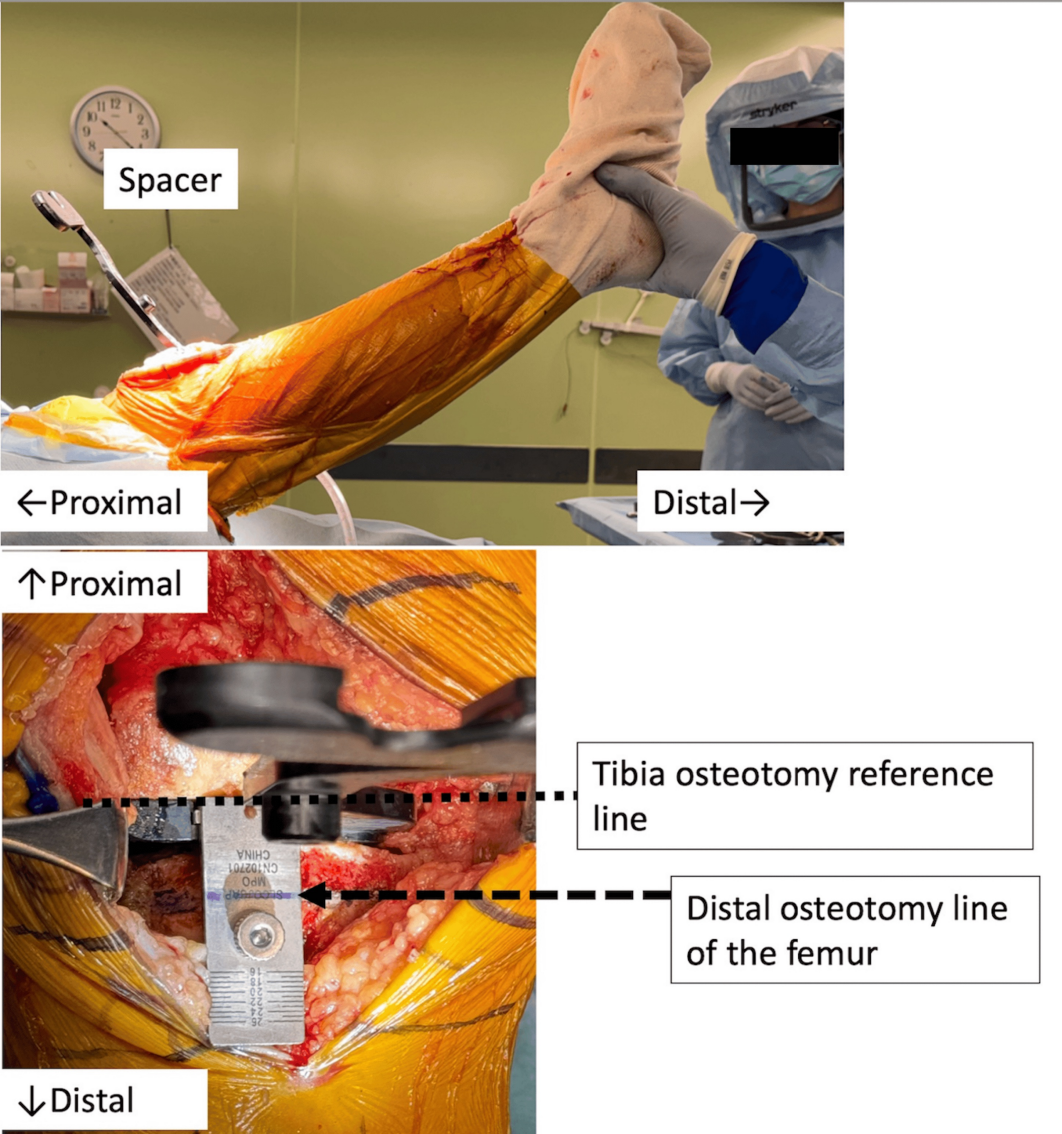
Heel lift technique The unique spacer is inserted to align with the distal femoral osteotomy surface, and the heel is lifted in extension. The line parallel to the distal femoral osteotomy plane and the osteotomy volume relative to the thickness of the implant can be visualized quantitatively simultaneously.

This is a novel technique for kinematic alignment tibial osteotomy. After the distal femoral osteotomy, soft tissue procedures such as anterior cruciate ligament reconstruction, posterior cruciate ligament reconstruction (if necessary), and meniscectomy are performed, and as many osteophytes as possible are removed. The varus-valgus alignment of the tibial osteotomy and the height of the osteotomy line can be confirmed by reference to the ligament balance obtained by inserting a unique rectangular spacer into the joint gap after distal femoral osteotomy to contact the distal femoral osteotomy surface. By lifting the heel during knee extension, the tibial osteotomy line and the amount of osteotomy required to restore the patient's individual joint line can be visualized using ligament taxis (Figure 3).

The principle of this method is similar to manual in-line traction [13,14]; however, it is highly reproducible because it requires only lifting the heel and holding it in extension, eliminating the need to be aware of the direction and force of the traction. This technique also allows the height of the osteotomy line to be checked simultaneously. The tibial osteotomy line obtained here is marked and referred to during subsequent tibial osteotomies.

Femoral Posterior Condyle Osteotomy

**Figure 4:**
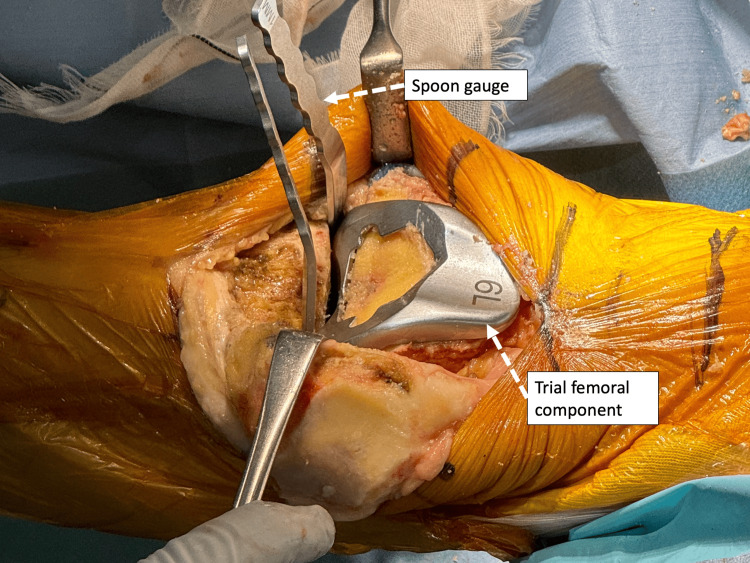
The medial and lateral gaps can be checked by a spoon gauge, respectively The gap can be checked by inserting a spoon gauge into the medial and lateral joint space with the trial femoral component in place. The gap information obtained can be used in the osteotomy volume and angle when using the double stylus for tibial osteotomy.

The femoral posterior condyle is osteotomized to match the thickness of the component, assuming there is no cartilage defect in the femoral posterior condyle. The femoral rotation should be osteotomized parallel to the posterior condyle axis. If there is a cartilage defect, the amount of osteotomy can be adjusted by modifying the angle of external rotation to accommodate the defect or by inserting a 1-2 mm spoon gauge into the paddle on the side where cartilage wear is noted. A posterior femoral sizing guide and a four-in-one resection guide are used to determine the femoral bone size. After the four-plane femoral osteotomy, the femoral trial component is placed such that the components match the native femoral groove. Patellar tracking is then checked, and if there are no problems, a peg hole is created, and the mediolateral position of the femur is determined. The gap is then checked by inserting a spoon gauge into the medial and lateral joint space with the femoral trial component in place. The obtained gap information can be used to confirm the osteotomy volume and angle when using a double stylus for tibial osteotomy (Figure 4).

Tibial Osteotomy

**Figure 5:**
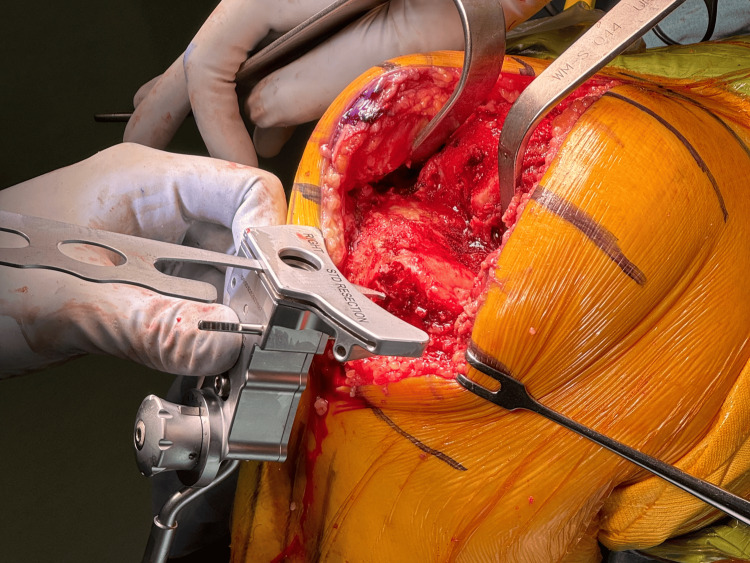
The tibial osteotomy height and varus-valgus alignment obtained with the heel lift technique can be easily checked with an angel wing gauge, even during tibial osteotomy

The concept of kinematic alignment is to regenerate the patient's joint surface shape through resurfacing and soft tissue respect [15,16]. Using the methods described above, femoral osteotomies can be performed relatively easily without CAS. However, for tibial osteotomies, the calipered method, which adjusts the angle based on the predicted articular surface shape, and the soft tissue-respected method, which refers to the angle obtained from ligament taxis by inline traction, are available for unrestricted KA. We also advocate the heel-lift technique as a new, highly reproducible, and visible method for confirming the angle and height of tibial osteotomy. The tibial osteotomy height and varus-valgus alignment obtained with the heel-lift technique can be easily checked with an angle wing gauge, even during tibial osteotomy (Figure 5).

**Figure 6:**
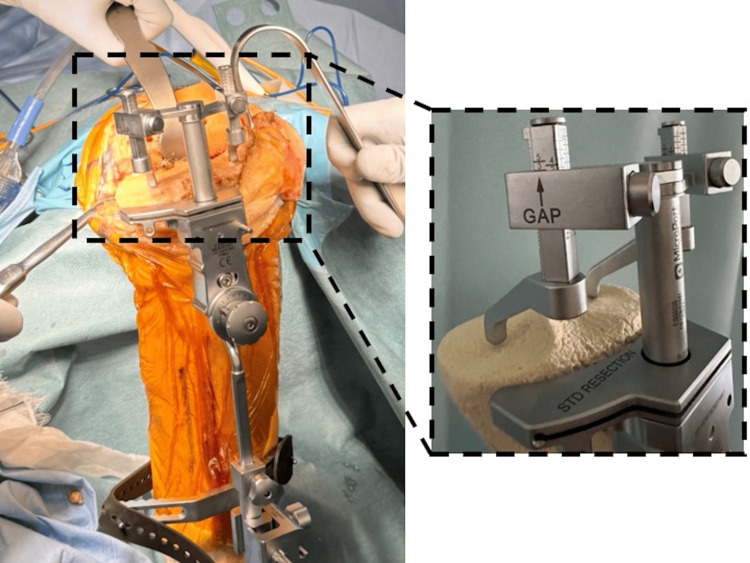
A stylus on the medial and lateral tibial articular surfaces The tibial extramedullary rod developed for the rKA-TKA can be used to confirm the amount of osteotomy by placing a stylus on the medial and lateral tibial articular surfaces, respectively, which is also helpful as an indicator of osteotomy amount and angle. The amount of osteotomy in the tibia can be checked using the medial and lateral gap information obtained from the spoon gauge as a reference. rKA-TKA: restricted kinematic-alignment total knee arthroplasty

Additionally, the tibial extramedullary rod developed for the rKA-TKA (MicroPort®, Memphis, USA) can be used to confirm the amount of osteotomy by placing a stylus on the medial and lateral tibial articular surfaces, respectively, which is also helpful as an indicator of osteotomy amount and angle (Figure 6).

**Figure 7:**
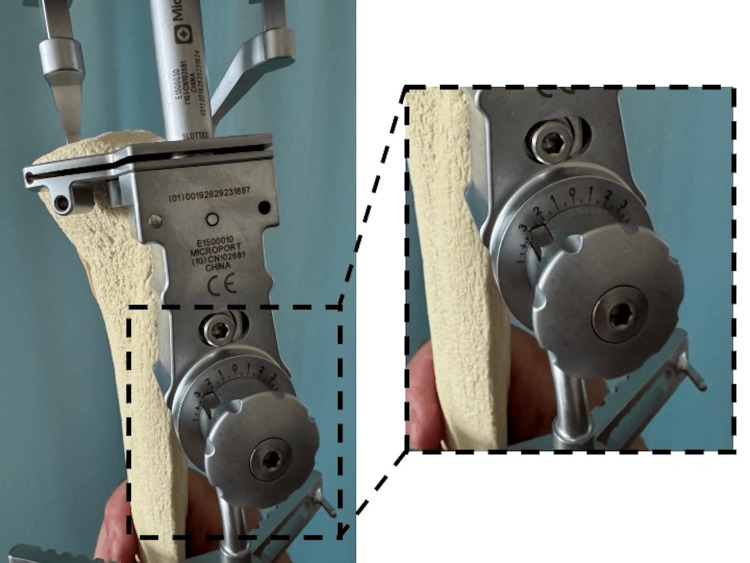
The extramedullary rod has an adjustable dial The extramedullary rod has an adjustable dial that can be changed in 0.5° increments up to 5° of varus and valgus, allowing restricted KA without CAS. KA: knee arthroplasty; CAS: computer-assisted surgery

The extramedullary rod has an adjustable dial that can be changed in 0.5° increments up to 5° of varus and valgus, allowing restricted KA without CAS (Figure 7).

**Figure 8:**
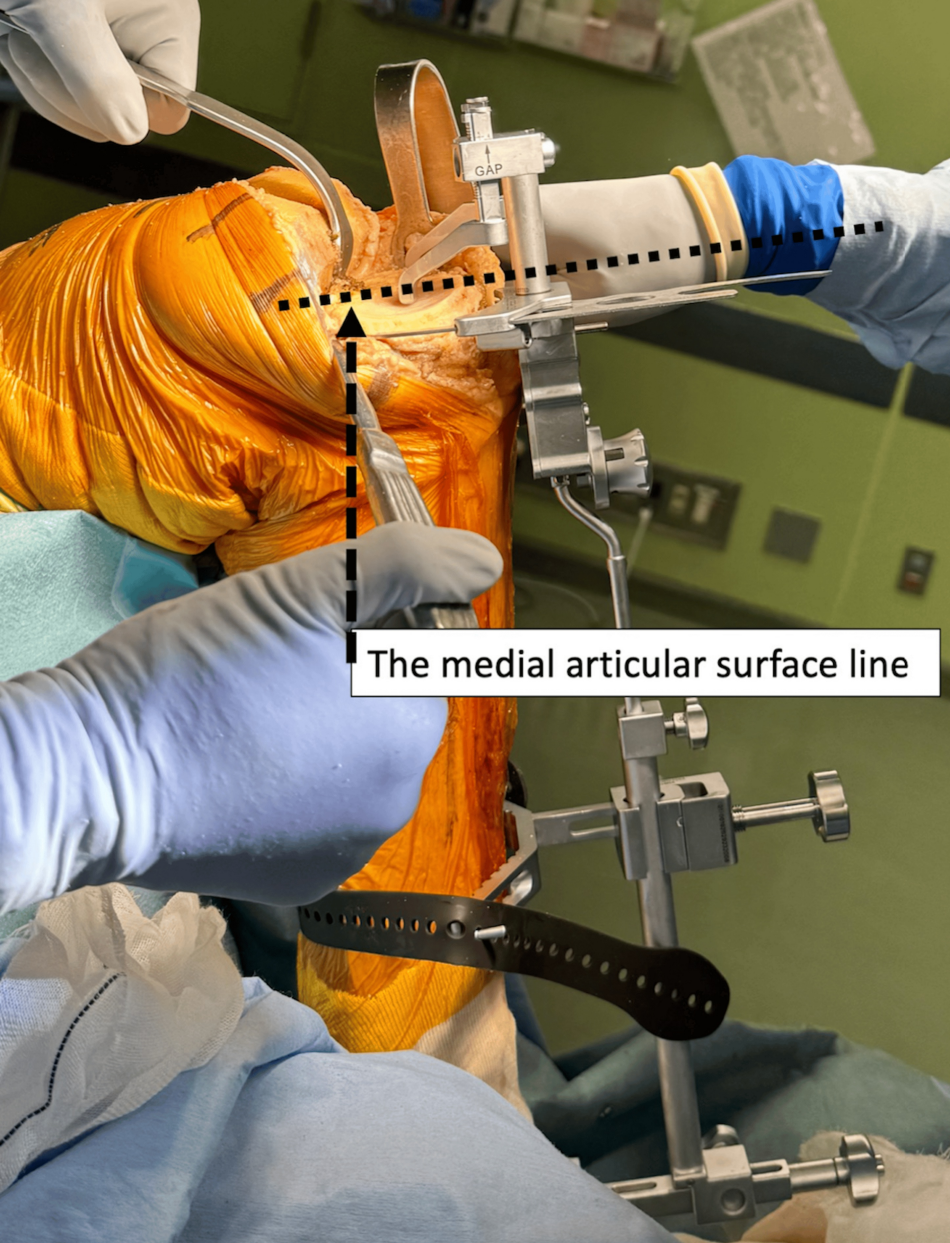
The posterior tilt angle is adjusted to be parallel to the medial articular surface using the angel wing

The posterior tilt angle is adjusted to be parallel to the medial articular surface using an angel wing. If a medial bone defect is present, the intercondylar ridge base is used as a reference (Figure 8). 

**Figure 9:**
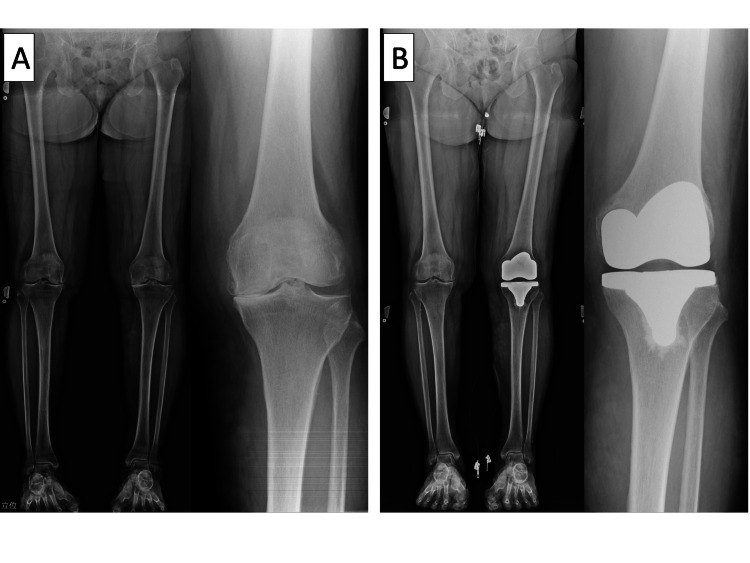
A) Standing lower limb X-rays before surgery and B) standing lower limb X-rays after surgery

At the end of the technical note, we have shared an X-ray of a case in which rKA-TKA was performed without CAS, with the tibial osteotomy angle and height determined by the heel lift technique (Figure 9).

## Discussion

Improving patient satisfaction, function, and long-term survival after TKA is a primary focus in orthopedic research. Traditional MA-TKA has long been considered crucial for enhancing long-term clinical outcomes and implant survival rates [17]. Studies indicate that KA-TKA, which restores the original joint line in individual patients, improves clinical outcomes without compromising implant survival [4,5,15]. KA-TKA aims to reestablish the original joint line orientation and constitutional alignment seen in preosteoarthritis. However, concerns exist that implant placement in extreme varus or valgus alignments may lead to early component loosening [17-20].

To address these concerns, Vendittoli proposed rKA-TKA for cases with extreme varus and valgus alignments [10]. In rKA-TKA, extreme alignments are avoided by finding an appropriate compromise within safe boundaries [9,21]. rKA-TKA was developed as an alternative to the unrestricted KA technique for patients with atypical knee anatomy. It offers a compromise that restores constitutional alignment in most cases, correcting extreme abnormal anatomy to an acceptable level with significantly less alteration to normal anatomy compared to MA. This positively impacts normal biomechanics, resulting in more balanced flexion and extension in the medial and lateral compartments [9,22]. Unlike traditional rKA-TKA procedures that require CAS tools such as robotics, navigation, and PSI, this report introduces a novel approach.

It is the first account of rKA-TKA using a conventional technique with a unique device that allows precise angle adjustment in 0.5° increments without CAS. We also introduce a new heel-lift technique, which we believe is a highly reproducible and surgeon-independent method for determining the angle and amount of tibial osteotomy required. These methods have significantly expanded the options for performing KA and rKA-TKA without CAS, making them accessible to any institution. 

Since the purpose of this study is to detail a new surgical technique, a limitation is the lack of data on surgical outcomes. Verification and reporting of data obtained using this surgical technique are necessary. Another limitation is the absence of quantitative evaluation of medial and lateral collateral ligament tension and the medial-lateral gap, a common issue in all TKA procedures not involving CAS. Even in robotic TKA, soft tissue balance is assessed manually, leading to considerable variability among surgeons. Moreover, navigation systems and robot-assisted technology have yet to resolve the issue of clinical satisfaction [23-26].

Therefore, we propose that this novel method of KA and rKA-TKA, which is not limited by cost or facility and can be performed with conventional methods, will be beneficial to all surgeons, regardless of the institution.

## Conclusions

Here, we present novel surgical devices and techniques for unrestricted KA and rKA-TKA, enabling quantitative angle adjustment without using CAS. Future studies should validate and report these results using this method. Patients who have undergone r-KA TKA using this technique have shown significant improvement, albeit in a short-term evaluation. Further detailed reports with imaging and clinical evaluations will be presented in the future.
